# Brain networks in eating disorders: a systematic review of graph theory studies

**DOI:** 10.1007/s40519-021-01172-x

**Published:** 2021-03-23

**Authors:** Enrico Collantoni, Francesco Alberti, Valentina Meregalli, Paolo Meneguzzo, Elena Tenconi, Angela Favaro

**Affiliations:** 1 Department of Neurosciences, University of Padua, Via Giustiniani, 2, 35128 Padua, Italy; 2 Padua Neuroscience Center, University of Padua, Padua, Italy

**Keywords:** Eating disorders, Anorexia nervosa, Bulimia nervosa, Brain networks, Neuroimaging, Graph theory

## Abstract

**Purpose:**

Recent evidence from neuroimaging research has shown that eating disorders (EDs) are characterized by alterations in interconnected neural systems, whose characteristics can be usefully described by connectomics tools. The present paper aimed to review the neuroimaging literature in EDs employing connectomic tools, and, specifically, graph theory analysis.

**Methods:**

A systematic review of the literature was conducted to identify studies employing graph theory analysis on patients with eating disorders published before the 22nd of June 2020.

**Results:**

Twelve studies were included in the systematic review. Ten of them address anorexia nervosa (AN) (AN = 199; acute AN = 85, weight recovered AN with acute diagnosis = 24; fully recovered AN = 90). The remaining two articles address patients with bulimia nervosa (BN) (BN = 48). Global and regional unbalance in segregation and integration properties were described in both disorders.

**Discussion:**

The literature concerning the use of connectomics tools in EDs evidenced the presence of alterations in the topological characteristics of brain networks at a global and at a regional level. Changes in local characteristics involve areas that have been demonstrated to be crucial in the neurobiology and pathophysiology of EDs. Regional imbalances in network properties seem to reflect on global patterns.

**Level of evidence:**

Level I, systematic review.

## Introduction

Eating disorders (EDs) are severe psychiatric conditions characterized by complex cognitive, psychopathological, and neurobiological underpinnings [1, 2]. In recent years, many efforts have been made to describe the neurobiological correlates of these disorders through functional and structural neuroimaging. As comprehensively described in recent reviews [3, 4], neuroimaging research in EDs indicates that their neural correlates could be better described as alterations in spatially distributed and interconnected neural systems rather than dysfunctions in single and spatially isolated areas. This evidence is consistent with the general observation that structural and functional brain alterations in psychiatric disorders can be better explained based on their covariance patterns rather than in terms of their localization [5]. A powerful tool to describe the organization of brain networks based on their covariance patterns has recently been offered by connectomic approaches, which evaluate the brain as a complex network and its alterations as modifications in the properties that govern its global or regional architecture [6]. One of the main advantages of using connectomics tools to evaluate brain structure and function is that they allow evaluating brain regions not as discrete and isolated elements but as components that interact with each other based on the topological characteristics of their connections.

The mathematical tool most commonly used in connectomics to describe the topological characteristics of brain areas within the brain is graph theory, a branch of mathematics that evaluates the properties and the interrelations between nodes and the edges connecting them [7]. Considering brain areas as nodes and their interrelations (functional or structural) as edges, graph theory can describe their topological properties employing different parameters, which can be divided according to their integration, segregation, or centrality characteristics. A correct balance between the integration and segregation properties of a network guarantees to properly couple its wiring cost with the ability to ensure communication between topologically distant areas [8]. The trade-off between integration and segregation characteristics of a network is summarized by the Small-World Index (SWI), which describes the position that a network occupies between in the continuum between regular and irregular systems [9]. Another key characteristic of the brain network is given by those nodes that are highly connected within the network, thus strongly contributing to its global structure and function, and that are generally referred to as network hubs [10]. The high connectivity of hub regions accounts for their elevated metabolic cost, which is supposed to be superior to one of more peripheric areas. However, the centrality of hubs is also hypothesized to account for a higher vulnerability to brain damages since dysfunctions in any brain region is more likely to spread through more connected nodes. Therefore, it is likely that brain disorders that implies high metabolic consequences like eating disorders could strongly impact on hubs configuration.

The segregation, integration, and centrality characteristics of a network are not stable during development and change profoundly according to the needs imposed by the different maturation phases and by the progressive recruitment of higher cognitive functions. Also, their balance varies profoundly due to the onset and progression of brain disorders, which often affects structures that require a higher functional cost, such as hubs. Eating disorders are of particular interest from this point of view since they generally emerge during adolescence or early adulthood, and are also often characterized by dramatic metabolic consequences [11]. For this reason, any alterations in the topological characteristics of the brain in these disorders must be carefully evaluated, as they can be explained both by changes that occur during neurodevelopmental trajectories and as consequences of the disorder progression. The use of multimodal imaging techniques can help in disentangling this complexity since alterations in different structural or functional indices can have different stability during developmental phases and a different sensitivity to environmental influences [12]. Moreover, longitudinal evaluation, the assessment of patients at different stages of the disorder (i.e., during acute phases or after recovery), as well as the estimation of graph properties alterations during severe and rapid weight loss, can be particularly useful for understanding how the properties that govern the network architecture vary over time and in different phases of the illness [13].

Alongside the possibility of evaluating the topological characteristics of the brain network, graph theory tools allow evaluating the strength of connectivity between nodes in case–control comparisons, through a network-based-statistics connectome approach [14]. Therefore, this approach does not measure the segregation or integration characteristics of a network but aims at identifying, in a between-group comparison, the presence of altered connectivity in subnetworks of two or more regions.

Overall, connectomics tools have been widely used in various psychiatric and neurological disorders and in eating disorders as well, allowing for deepening their neurobiological underpinnings. Therefore, in the present review, we are aimed at highlighting recent findings reported in MRI-based connectomic studies of eating disorders, which employed graph theory tools.

## Methods

### Literature search

A systematic review was performed in accordance with the Preferred Reporting Items for Systematic reviews and Meta-Analyses (PRISMA) statement [15]. A literature search was conducted on the 22nd of June 2020 on two online digital archives, PubMed and SCOPUS, to identify fitting papers published from inception up to June 2020.

The following search key was used: “graph” AND (“anorexia nervosa” OR “bulimia nervosa” OR “eating disorders”). The reference lists of relevant articles were examined as well in order to identify further papers of interest.

### Study selection

After the literature search, two authors (FA and EC) independently screened the title/abstract studies' list and then proceeded with a full-text assessment of the remaining papers.

Ultimately, in the review were only included peer-reviewed studies that respected the following inclusion criteria: (i) including patients diagnosed with EDs according to DSM-IV or DSM-5 criteria; (ii) including a control group of healthy participants; (iii) performing graph analysis on neuroimaging data. Previous reviews and meta-analyses were read in full text to identify further studies. Only studies written in English were considered.

A third author (VM) was called upon to solve any conflict that emerged in the study selection process.

### Data extraction and quality assessment

During the full-text assessment, the following information was extracted into a standardized Excel sheet: (i) study population characteristics (e.g., sample size, demographics, diagnostic criteria and subtype of ED, and number of medicated participants); (ii) neuroimaging methods and additional measures (e.g., BMI, neuropsychological tests administered, and cognitive tasks performed); (iii) graph analysis characteristics (e.g., type of data used, main features of the graphs, and kinds analyses performed); (iv) main results of the analyses.

The methodological quality of the studies was assessed using the protocol proposed by Olivo et al. [16], derived from the guidelines for reliable neuroimaging research in EDs outlined by Frank et al. [17]. The protocol includes 31 items divided in 6 categories (for full list see Olivo et al. [16]): (i) development, demographic data, and illness state; (ii) effects of exercise, hydration status, binge eating and purging, and malnutrition; (iii) stage of treatment; (iv) hormonal effects; (v) comorbidity and medication; (vi) technical and statistical considerations, and study design. Items concerning longitudinal assessments (i.e., #6) and hormonal measures (i.e., #21 and #22) were excluded as they were not applicable to any of the included studies. Additionally, item #28, concerning stimuli selection for task-based fMRI, was also excluded as it was applicable to only one study. The papers were evaluated by assigning them a score between 0 and 1 for each of the remaining 27 items and multiplying it by an index of the importance of the item [13]: essential (3); strongly desirable (2); desirable (1). The sum of the obtained values represented the QA score of the publication, which is comprised between 0 and 68.5.

## Results

**Fig. 1:**
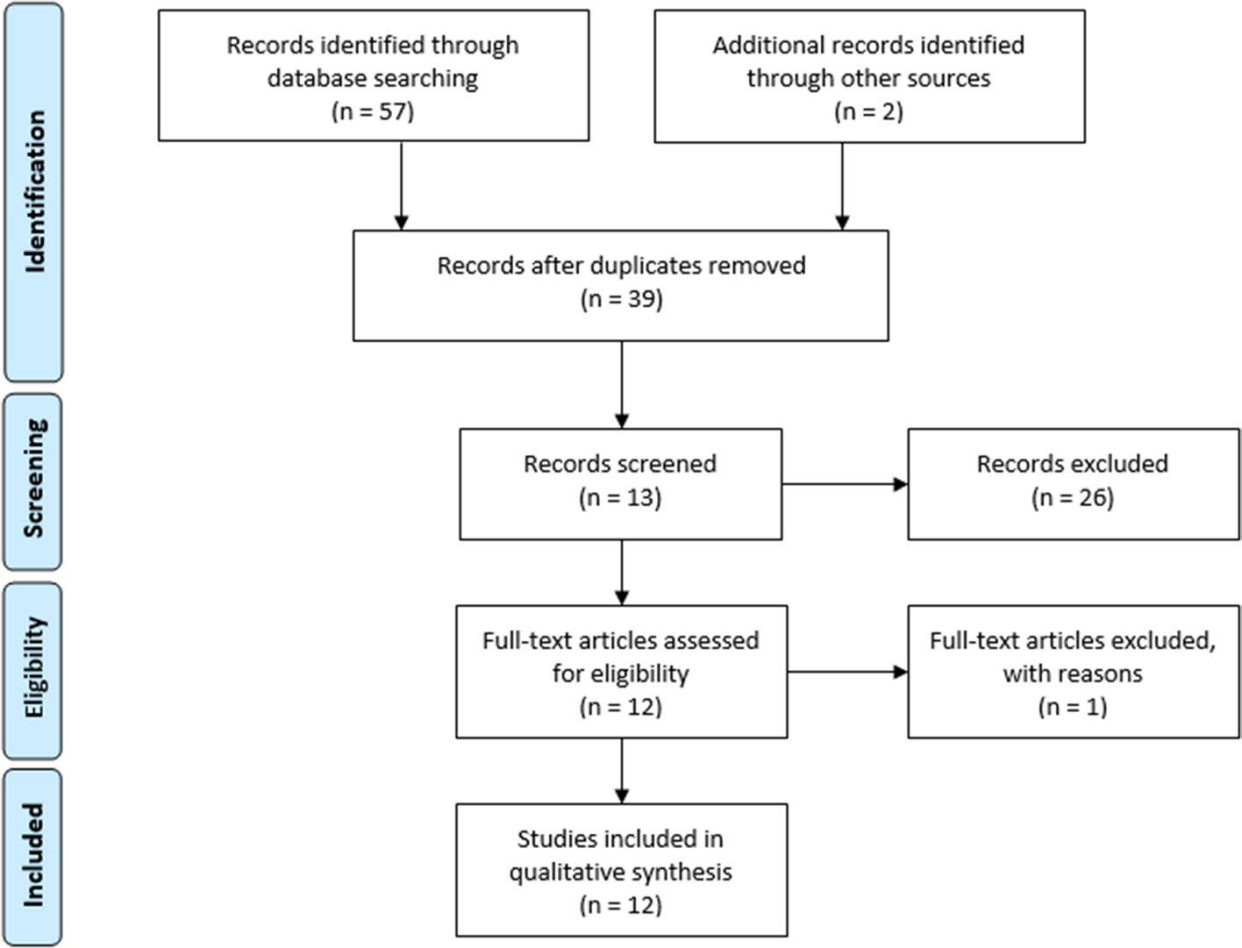
PRISMA flow diagram

As summarized in Fig. 1, the database search produced 37 results (after discarding doubles), and two articles were identified through other sources [18]. Out of these studies, 26 were excluded based on title or abstract as they either did not include an experimental group of ED patients (acute or recovered) or did not apply graph theory analyses to neuroimaging data. After the full-text examination of the studies, one more was excluded since it did not respect all inclusion criteria, while the remaining 12 were included in the systematic review. Ten of them address AN, counting 199 patients overall: 85 with acute diagnosis (AN-a), 24 weight-recovered with acute diagnosis (AN-wr), and 90 fully recovered (AN-r). The remaining two articles, instead, address Bulimia Nervosa (BN) and are based on a single sample of 48 patients with acute diagnosis (BN-a). Tables 1 and 2 summarize, respectively, the main demographic information of the participants, and the core methodological characteristics and results of all the included studies.

**Table 1:** Main participant demographic and clinical data of the studies included in the systematic review

Study	Nationality	Group	*N* (f/m)	Age: years (SD)	Medicated	Diagnostic creteria	BMI: mean (SD)	Illness duration: months (SD)
Functional connectivity networks								
Collantoni et al. 2019 [19]	Italian	AN-a	36 (36/0)	26.0 (7.0)	13/36	DSM-5	15.8 (1.8)	73.3 (79.2)
		HC	38 (38/0)	25.3 (6.3)	None	n/a	21.7 (2.9)	n/a
Geisler et al. 2016 [21]	German	AN-a	35 (35/0)	16.1 (2.6)	None	DSM-IV	14.8 (1.3)	n/a
		HC	35 (35/0)	16.2 (2.6)	None	n/a	20.8 (2.7)	n/a
Geisler et al. 2019 [28]	German	AN-r	55(55/0)	22.4 (3.3)	2/55	DSM-IV	20.7 (1.7)	n/a
		HC	55(55/0)	22.4 (3.3)	None	n/a	21.7 (2.1)	n/a
Kullmann et al. 2014 [22]	German	AN-a	12(12/0)	23.3 (4.7)	None	DSM-IV	15.5 (1.5)	n/a
		HC	14(14/0)	24.6 (2.9)	None	n/a	21.4 (1.5)	n/a
		HC (athletes)	12(12/0)	24.1 (3.2)	None	n/a	22.0 (1.9)	n/a
Lord et al. 2016 [23]	German	AN-a	35 (35/0)	16.1 (2.6)	None	DSM-IV	14.8 (1.3)	n/a
		HC	35 (35/0)	16.2 (2.6)	None	n/a	20.8 (2.7)	n/a
Ehrlich et al. 2015 [20]	German	AN-a	35 (35/0)	16.1 (2.6)	None	DSM-IV	14.8 (1.3)	n/a
		HC	35 (35/0)	16.2 (2.6)	None		20.8 (2.7)	n/a
Gaudio et al. 2018 [28]	Italian	AN-r	15(15/0)	15.7 (1.7)	None	DSM-IV	16.1 (1.2)	4.0 (1.8)
		HC	15(15/0)	16.1 (1.4)	None	n/a	21.6 (2.4)	n/a
Wang et al. 2017 [29]	Chinese	BN-a	44 (44/0)	22.0 (3.4)	None	DSM-IV	21.0 (2.6)	24.0(15.6)
		HC	44 (44/0)	23.1 (3.4)	None	n/a	20.5 (1.4	n/a
Structural connectivity networks								
Vaughn et al. 2019 [26]	US	AN-wr	24 (23/1)	21.0 (5.0)	None	DSM-IV	20.0 (2.0)	72.0 (63.0)
		BDD-a	29 (26/3)	23.0 (5.0)	None	DSM-IV	22.0 (3.0)	118.0 (70.0)
		HC	31 (25/6)	21.0 (5.0)	None	n/a	22.0 (3.0)	n/a
Zhang et al. 2016 [25]	US	AN-wr	24 (23/1)	21.3 (4.5)	None	DSM-IV	20.1 (1.5)	72.2 (63.0)
		BDD	29 (25/4)	23.2 (5.0)	None	DSM-IV	21.8 (2.8)	16.8 (69.9)
		HC	31 (25/6)	20.9 (3.9)	None	n/a	22.0 (3.0)	n/a
Wang et al. 2019 [30]	Chinese	BN-a	48 (48/0)	22.0 (3.4)	None	DSM-IV	21.0 (2.6)	24.0(15.6)
		HC	44 (44/0)	23.1 (3.4)	None	n/a	20.5 (1.4	n/a
Structural covariance networks								
Collantoni et al. 2019 [19]	Italian	AN-a	38 (38/0)	26.1 (7.2)	14/38	DSM-5	15.8 (1.8)	78.6 (81.3)
		AN-r	20 (20/0)	26.3 (7.1)	4/20	DSM-5	19.6 (1.6)	45.7 (65.0)
		HC	38 (38/0)	25.3 (6.3)	None	n/a	21.7 (2.9)	n/a

*BMI* body mass index, *SD* standard, *ANa* patients with acute anorexia nervosa, *ANwr* weight-recovered patients with anorexia nervosa, *ANr* recovered patients with anorexia nervosa, *HC* healthy controls

**Table 2:** Main methodological characteristics of the studies included in the systematic review

Study	Genetic, physiological, and psychological measures	Imaging protocol	Connectivity measures	Graph features	Graph measures	Comparison	Graph analysis results	QA
Functional connectivity networks								
Collantoni et al. (2019) [19]	SCL-90-R, EDI-2, STAI WCST, ROCF, IGT 5-HTTLPR genotyping	fMRI: Resting state	FC	Type: BU Nodes: 148 Threshold: 0.1–0.5	Topological metrics	AN-a vs HC	Globally, AN-a showed lower CC For hubs: based on betweenness the left SFG hub lacked in AN-a, while ACC was a hub only in AN-a; based on degree higher values were found in ACC and MFG for AN-a, and in PHG, left transverse frontopolar gyrus, and right posterior lateral sulcus for HC	39
						5-HTTLPR: S vs L	In AN-a the S genotype correlates with lower SWI and modularity; in HC it correlates with higher modularity	
Geisler et al. (2016) [21]	EDI-2, BDI-2, SCL-90-R	fMRI: Resting state	FC	Type: BU Nodes: 160 Density: 10–30%	Topological metrics	AN-r vs HC	Both group showed a small-world organization Globally, AN-a had higher CPL and assortativity Locally, AN-a showed: lower CPL in left middle insula, right posterior insula, and bilateral thalamus; lower strength in left middle insula, right posterior insula, and left thalamus; lower degree in left middle insula and right posterior insula; higher LEGE in right posterior occipital cortex; higher LE in right anterior PFC	48.75
Geisler et al. (2019) [28]	EDI-2, BDI-2, SCL-90-R Plasma leptin	fMRI: Resting state	FC	Type: BU Nodes: 160 Density: 10–30%	Topological metrics	AN-r vs HC	AN-r have higher assortativity and lower SWI and CC	43.75
					SVC (local graph metrics)	SVC	AN-r/HC classification reached 70.4% accuracy based on nodal measures	
					NBS	BN-a > HC HC > BN-a	No significant differences	
Kullmann et al. (2014) [22]	EDI-2, BAS/BIS, CES Plasma leptin	fMRI: Resting state	FC	Type: WU Nodes: 160	Degree centrality	AN-a vs HC	AN-a showed lower degree centrality of IFG	31
					Effective connectivity	AN-a vs HC	In AN-a effective connectivity is reduced from right IFG to mid-cingulum and increased from OFC to right IFG, and from the insula to left IFG	
Lord et al. (2016) [23]		fMRI: Resting state	FC	Type: WU Nodes: 90 Density: 10–30%	Topological metrics	AN-a vs HC	Globally, AN-a showed higher CPL and assortativity (in both parcellations) Based on AAL AN-a show: altered path length in the precentral gyrus and left postcentral gyrus; altered LEGE in the left calcarine cortex. Based on Dosenbach AN-a show: altered pathlength in right precentral gyrus, thalamus, and posterior insula; altered LEGE in the right posterior occipital cortex; altered degree in the left mid and posterior insula; altered strength in the left thalamus, mid insula, and posterior insula	25.5
				Type: WU Nodes: 160 Density: 10–30%	NBS	BN-a > HC HC > BN-a	AN-a hypoconnectivity network (overlapping across parcellations) includes posterior insula, thalamus, and right fusiform gyrus	
Ehrlich et al. (2015) [20]	EDI-2, WAIS/WISC, EHI Plasma leptin	fMRI: Resting state	FC	Type: WU Nodes: 104	NBS	BN-a > HC HC > BN-a	BN-a hypoconnectivity network including left amygdala and thalamus, right fusiform gyrus, and bilateral putamen and posterior insula	32.75
					Intranodal Homogeneity (KCC)	AN-a vs HC	No significant differences	
Gaudio et al. (2018) [28]	EDI-2, BDI, STAI	fMRI: Resting state	FC	Type: WU Nodes: 128	NBS	HC > AN-r	AN-r hypoconnectivity network includes bilateral rostral ACC, right superior occipital cortex, left paracentral lobule, cerebellum (lobule X), posterior insula, and medial OFC	42.5
					Intranodal Homogeneity (KCC)	AN-r vs HC	No significant differences	
Wang et al. (2017) [29]	EDI-1, HAMD-17, HAM-A	fMRI: Resting state	FC	Type: BU Nodes: 90 Density: 10–34%	Topological metrics	BN-a vs HC	Globally, CC and CPL was higher in BN-a Locally, in BN-a strength was higher in primary sensorimotor and unimodal visual association cortices, and lower in medial OFC, MTL, left insula, left amygdala, left putamen, and left thalamus	27.5
					NBS	BN-a > HC HC > BN-a	BN-a showed: hyperconnectivity among primary sensorimotor, unimodal association, and multimodal association networks; and hypoconnectivity among caudate, putamen, thalamus, amygdala, hyppocampus, OFC, ACC, and PHG	
Structural connectivity networks								
Vaughn et al. (2019) [26]	MINI, YBC-EDS, EDE, BABS, HM (HAM-A + MADRS)	DWI	FACT	Type: WU Nodes: 87	Normalized path length	Classification:HC/AN-BDD	leave-one-out: 89%, PPV: 89%, NPV: 84%, AUC-ROC: 93% Results significantly driven by association between low HM scores and HC	32
						Classification:AN/BDD	leave-one-out: 74%, AN: 78%, BDD: 71%, AUC-ROC: 67% Driven by association between high NPL and AN, and between BABS scores and BDD	
						HC/AN-BDD + AN/BDD	leave-one-out: 76%, AN: 63%, BDD: 76%, HC: 84%	
Zhang et al. (2016) [25]	MINI, HAM-A, MADRS, BABS, BDD-YBOCS, EDE	DWI	FACT	Type: WU Nodes: 87	Path length associated community estimation	HC vs AN-wr vs BDD	Identified one differing network In HC: right caudate, pallidum, Nacc, posterior ACC, anterior ACC, and PCC In AN-wr: right caudate, Nacc, rostral ACC, lateral and medial OFC, frontal pole In BDD: right caudate, pallidum, anterior ACC, PCC, medial OFC	29
					Topological metrics	HC vs AN-wr vs BDD	NPL was significantly different among groups and it was higher in AN-wr compared to BDD and HC	
					Modularity metric Q		No significant differences	
					Scaled inclusivity		No significant differences	
Wang et al. (2019) [30]	MINI, EDI-1, HAMD, HAM-A	DWI	FACT	Type: BU Nodes: 90	Topological metrics	BN-a vs HC	BN-a patients showed: increased strength in left superior OFC, left ITG, left insula, left hippocampus, left PHG, left thalamus; reduced strength in left ACC and right precuneus; increased betweenness in left superior medial OFC, left ACC, left STG, left superior temporal pole, left precuneus, right fusiform gyrus, left insula, left PHG, left putamen, right pallidum, left thalamus, right amygdala; reduced betweenness in right IFG, right superior OFC, left fusiform gyrus, and right insula; increased LE in left superior OFC, left STG, left superior temporal pole, left thalamus, and left amygdala; reduced LE in right precentral gyrus and precuneus; reduced GE in OFC, gyrus rectus, insula, putamen, pallidum, amygdala, precentral gyrus, postcentral gyrus, supramarginal gyrus, precuneus, fusiform gyrus	27.5
					NBS	BN-a > HC HC > BN-a	BN-a showed: a hyperconnectivity network including OFC, ACC, insula, caudate, thalamus, temporal-occipital cortex, and PHG; a hypoconnectivity network including IFG, insula, and temporal cortex	
Structural covariance networks								
Collantoni et al. (2019) [19]	SCL-90-R, EDI-2, EHI AN-a 3 years follow-up	MRI	LGI	Type: BU Nodes: 148 Density: 10–50%	Topological metrics	AN-a vs HC	AN-a show higher SWI	39
						AN-r vs HC	No significant difference	
						good vs poor outcome AN-a	Poor outcome patients show higher CC and normalized degree in the right insula	
			CT	Type: BU Nodes: 148 Density: 10–50%	Topological metrics	AN-a vs HC	AN-a show higher mean LE, CC, modularity, SWI, and lower GE	
						AN-r vs HC	No significant differences	
						good vs poor outcome AN-a	Good outcome patients have higher degree in left IFG, poor outcome ones have higher clustering in the left IFG	

*ACC* Anterior cingulate cortex, *ANa* patients with acute anorexia nervosa, *ANwr* weight-recovered patients with anorexia nervosa, *ANr* recovered patients with anorexia nervosa, *HC* healthy controls, *BABS* Brown assessment of beliefs scale, *BDD-a* Body dysmorphic disorder (acute), *BDD-YBOCS* BDD version of the Yale-Brown obsessive–compulsive scale, *BDI* Beck's depression inventory, *BDI-2* Beck's depression inventory-2, *BMI* body mass index, *BN* bulimia nervosa, *BU* binary undirected, *CC* clustering coefficient, *CCI* Central coherence index, *CES* Commitment to exercise scale, *CPL* characteristic path length, *CT* cortical thickness, *DSM-5* Diagnostic and statistical manual of mental disorders-5th edition, *DSM-IV* Diagnostic and statistical manual of mental disorders-4th edition, *DTI* diffusion tensor imaging, *EDE* Eating disorder evaluation, *EDI-1* Eating disorder inventory 1, *EDI-2* Eating disorder inventory 2, *EHI* Edinburgh handedness inventory, *FC* Functional connectivity, *fMRI* Functional magnetic resonance imaging, *GE* Global efficiency, *HAM-A* Hamilton anxiety rating scale, *HAMD-17* Hamilton rating scale for depression, *HC* healthy control, *IFG* inferior frontal gyrus, *IGT* iowa gambling task, *ITG* inferior temporal gyrus, *KCC* Kendall's coefficient concordance, *LE* local efficiency, *LEGE* normalized local efficiency, *LGI* local gyrification index, *MADRS* Montgomery-Asberg depression scale, *MFG* middle frontal gyrus, *MINI* Mini international neuropsychiatric interview, *MTL* mediotemporal lobe, *NBS* network-based statistics, *NPL* normalized path length, *NPV* negative predictive value, *OFC* orbitofrontal cortex, *PHG* parahippocampal gyrus, *PPV* positive predictive value, *QA* quality assessment, *ROCF* Rey–Osterrieth complex figure test, *SCL-90-R* Symptom checklist-90-revised, *SFG* superior frontal gyrus, *sMRI* structural magnetic resonance imaging, *SPL* superior parietal lobule, *STAI* State-trait anxiety inventory, *STG* superior temporal gyrus, *SVC* support vector classifier, SWI small-worldness index, TIB Brief intelligence test, *WAIS-R* Wechsler adult intelligence scale-revised, *WCST* Wisconsin card sorting test, *WISC* Wechsler intelligence scale for children, *WU* weighted undirected, *YBS-EDS* Yale-Brown-Cornell eating disorder scale

### Functional connectivity graph analysis on acute patients with AN

Most studies including AN-a participants (i.e., five) analyzed resting-state functional connectivity (FC) graphs [19–23]. In three instances, global topological features resulted altered. Two of these studies [21, 23] have found higher characteristic path length (CPL) and assortativity in AN-a compared to healthy controls (HC). Among these, a study by Lord and colleagues (2016) also proved such difference in assortativity values to be consistent across two different parcellation atlases: AAL and Dosenbach [23]. A third paper [19], instead, has found a lower clustering coefficient (CC) in the AN-a group and a different hub nodes distribution between patients and controls. From this study emerged that, based on betweenness centrality, the anterior cingulate cortex (ACC) represented a hub only in AN-a and the superior frontal gyrus only in HC. Referring to degree centrality, instead, ACC and middle frontal gyrus (MFG) displayed higher values in AN-a, while left transverse frontopolar gyrus and right posterior-lateral sulcus were hubs only in HC. Additionally, the authors also compared AN-a participants based on the 5-HTTLPR polymorphisms and found that, while in HC the short variant of this gene was associated with higher modularity, in AN-a it correlated with lower SWI and modularity [19].

Furthermore, nodal topological features of AN-a graphs, instead, were altered in three studies [21–23]. The first study [21] found: lower CPL, strength, and degree in the insula (left middle and right posterior); lower CPL and strength in the thalamus; increased normalized local efficiency (LEGE) in the posterior occipital cortex; and increased local efficiency (LE) in the right anterior prefrontal cortex (PFC). The second study [22], instead, assessed only degree centrality, which was significantly reduced in AN-a patients in the inferior frontal gyrus (IFG). In the last study [23], Lord and colleagues (2016) reported that a consistent alteration of path length and LEGE across the two tested parcellations, albeit affecting different nodes based on the atlas (see Table 2). Only the path length of the right precentral gyrus was altered in both graphs.

Moreover, two studies performed a network-based analysis (NBS) of FC graphs. The first is the already-mentioned study by Lord and colleagues, who evidenced the presence of hypoconnectivity networks in AN-a patients using both parcellations [23]. These networks overlapped in the posterior insula, thalamus, and right fusiform gyrus (FFG) across atlases. The second study, by Ehrlich and collaborators, identified a similar hypoconnectivity network that also encompassed posterior insula, left thalamus, and right FFG together with putamen and left amygdala [20]. Neither of the articles has found evidence of any hyperconnectivity network in AN-a.

### Structural connectivity graph analysis on acute patients with AN

Among the studies on patients with acute AN, three used structure-based graphs. The first [24] built separate graphs based on two morphological measures: cortical thickness (CT) and local gyrification index (LGI). Analyzing the CT graph, AN-a showed lower global efficiency (GE) and higher LE, CC, modularity, and small-world index (SWI). The LGI graph, instead, only displayed increased SWI in AN-a. Moreover, comparing poor- and good-outcome patients, the authors found that based on CT poor outcome was correlated with higher clustering in the left IFG, while the recovery was associated with a higher degree in this same region and, based on LGI, with higher CC and insular NPL. The remaining two studies, instead, built structural connectivity graphs based on DTI. Both are based on AN-wr patients and include a group of acute body dysmorphic disorder patients (BDD-a). One of the two [25] has found a significantly higher NPL in AN-wr than in both BDD-a and HC participants. Additionally, among other modularity metrics (see Table 2), the authors performed a path-length-associated community estimation (PLACE) and identified a module of nodes that varied significantly between patients and controls. In HC, this community comprised the right caudate, right accumbens, right ACC, right posterior cingulate, and right pallidum, while in AN-wr it included the right caudate, right accumbens, right rostral ACC, right medial and lateral OFC, and right frontal pole (the BDD-a module shared some elements with each of other groups, see Table 2 for details). Based on these results, the second study [26] implemented a two-step machine learning model using DTI-based NPL as a feature in conjunction with other measures (i.e. task-related FC, anxiety, depression, and insight). The model successfully discriminated AN-wr and BDD-a from HC first (89.0% accuracy), and then AN-wr from BDD-a (74.0% accuracy) with a significant association between higher NPL and anorexic participants (and between poorer insight and BDD-a).

### Functional connectivity graph analysis on recovered AN patients

Of the three studies on patients that fully recovered from AN, two analyzed resting-state FC graphs and performed an NBS analysis. However, while one identified a hypoconnectivity network in AN-r that included rostral ACC, right superior occipital cortex, left paracentral lobule, left posterior insula, left medial OFC, and left lobule X of the cerebellum [27], the other did not find any altered network in AN-r [28]. The second study, however, analyzed topological graph metrics as well and has found higher assortativity and lower SWI and CC in AN-r. The Authors also implemented a machine learning model that was able to classify AN-r and HC participants with 70.4% accuracy using as features the nodal graph metrics [28].

### Structural connectivity graph analysis on recovered AN patients

Only one study based on structural imaging included an AN-r group [19] which, however, did not find any evidence of topological differences between recovered patients and healthy participants neither in the CT nor in the LGI graphs. Nonetheless, this result might have been conditioned by the small numerosity of the AN-r group included by the study which may have affected the power of the analyses run on this sample.

### Functional connectivity graph analysis on acute patients with BN

So far, only one study [29] has applied graph analysis to resting-state FC in BN-a patients, and its Authors found overall higher clustering and path length in BN-a and altered nodal strength in several vertices. Indeed, higher strength was found in the precuneus and in multiple nodes belonging to the primary sensorimotor and visual association cortices. Medial regions of the OFC and temporal lobe together with the insula and several subcortical structures, inversely, showed reduced strength in bulimic participants. Furthermore, from NBS analyses emerged a hyperconnectivity network encompassing the primary sensorimotor, unimodal association and polymodal association systems, and a hypoconnectivity network comprising limbic and paralimbic cortices as well as various subcortical regions.

### Structural connectivity graph analysis on acute patients with BN

The only other study found in literature addressing BN [30], instead, applied graph analysis to DTI data finding an asymmetric alteration of nodal metrics in bulimic patients compared to controls. A large array of left lateralized regions belonging to the mesocorticolimbic reward system, lateral Occipito-temporal cortex, and precuneus showed higher strength, betweenness, and LE. On the contrary, right-sided nodes of the mesocorticolimbic system, somatosensory, and visuospatial networks displayed reduced GE and betweenness. Additionally, NBS analyses identified: a hyperconnectivity network within the reward circuitry with a (largely involving the OFC) and between OF and occipitotemporal regions. Hypoconnectivity, instead, was found between the IFG and the lateral temporal cortex.

## Discussion

From the present review emerged that connectomic research in eating disorders is somewhat limited and employs a heterogeneous array of methodological approaches, making it difficult to draw direct comparisons or give a univocal interpretation of the results. Nonetheless, certain findings show noteworthy patterns.

Most papers examined brain networks of patients with AN, both acute and recovered. Overall, the studies report fairly consistent results in the resting state and DTI networks, which diverge instead from cortical structure ones, probably reflecting different disorder-related consequences on these systems. On a global level, AN seems associated with a longer path length: the increased CPL found in FC graphs of acutely underweight patients [21, 23] is mirrored by the increased NPL found in DTI graphs of weight recovered ones [25]. An increased path length indicates a less efficient transfer of information across the network [31], which is believed to affect integration processes [32]. The presence of reduced efficiency in brain network architecture in patients with acute AN is also supported by the evidence of a reduced clustering coefficient [19, 28]. In fact, lower clusterization, paired with higher path length, could bolster a more random and less small-world like network organization. Nevertheless, it should be evidenced that no study to date found reduced SWI in patients with acute AN. Overall, these findings help explain the higher assortativity observed in patients with AN [21, 23], which represents the increased nodes’ tendency to link with other nodes of a similar degree. Higher assortativity is generally a cost-efficient feature as the network is more likely to use a limited core of high-degree interconnected nodes acting as connector hubs [33] while other nodes link in specialized clusters. However, since a low clustering indicates a reduction of short-range connections, it could be speculated that the assortativity index is brought up by a decrease of low- to high-degree (or cluster to hub) links, as suggested by the increased path length and the altered hub distribution of individuals with AN [19].

This view is somewhat in line with the finding of decreased CPL, degree, and strength in the thalamus and posterior insula [21, 23], as these areas are, respectively, a core sensory relay station and a region with high centrality values in the healthy brain [10]. Both these regions are also part of the hypoconnectivity networks detected in functional connectivity graphs of AN [20, 23], further suggesting a disruption of the integrity of the thalamo-insular subnetwork, which was proposed to be fundamental for the internal representation of the physiological body state [34]. Moreover, the reduced integration characteristics of these two areas, which are fundamental in conveying ascending information to the rest of the cortex, could support the already proposed presence of an imbalance between top-down and bottom-up stimuli representations in the pathophysiology of AN [35, 36].

This network disruption might be a consequence of energetic and metabolic imbalances caused by malnutrition. Nonetheless, structural NPL is high in weight-recovered patients [25], and the increase of CC and assortativity [19, 28] persists in recovered participants, who also display reduced small-worldness. Therefore, such alterations could also be trait markers of AN or scars that persist after recovery. Longitudinal observations are needed to clarify this point.

Interestingly, the morphological graph analyses yielded partially opposite results, with acute patients displaying an increased small worldness in both CT- and LGI-based networks [24]. Moreover, CT graphs display high CC, modularity, and LE that indicate a higher level of segregation among clusters, which is probably what makes the network more small-world-like, thus theoretically more efficient. However, the decrease in GE is also a sign that the cost-efficiency of routing between distant structures is reduced at the expense of integration.

Concerning these results, however, it is important to note that although a correspondence has been established between structural covariance networks and functional/structural connectivity networks in healthy individuals [37, 38], this notion might not hold true in acute AN due to the impact of malnourishment and dehydration on brain morphology [1]. The observed alterations in structural covariance networks may be dictated, at least partly, by transient reductions in cortical thickness and complexity rather than by connectivity alterations [39]. Reductions in thickness and gyrification indexes often emerge soon in acute AN [40–42] and rapidly recover with weight restoration [43, 44]; therefore, they are unlikely to reflect changes in structural–functional connectivity. Taken together, these observations support the importance of multimodal imaging analysis on the study of brain alterations in AN, to deepen the neurobiological effects of different pathogenic mechanisms. For example, it is likely that cortical structures are more influenced by the acute effects of malnutrition, while functional and white matter connectivity patterns are more affected by other processes.

As for studies on BN, the topological characteristics of functional and structural graphs tend to diverge as many regions that had reduced strength in the first had increased strength in the second [29, 30]. Moreover, the finding of increased CC and CPL of FC graphs was not replicated based on DTI. Results of NBS analyses vary greatly between graph types. Many of the same limbic and paralimbic regions participating in the mesocorticolimbic reward system appeared to be hypoconnected in functional networks and hyperconnected in structural ones. Additionally, the extremely limited number of studies and the fact that they all analyzed the same sample of participants make these results even harder to interpret and generalize. Thus, the relationship between the structural and functional alterations in the brain networks of BN patients is still unclear and needs further investigation.

In conclusion, the present review shows that the literature concerning the use of graph theory in the neuroimaging research of EDs is still in its infancy. Given the potentialities that these research techniques have in addressing important issues in eating disorders research, further studies that can overcome the limits imposed by low sample sizes and by the absence of longitudinal analysis are needed.

Overall, connectomic studies in EDs evidenced the presence of an imbalance between segregation and integration properties in functional and in structural networks. These alterations, detected in brain areas that showed to be crucial in AN and BN neurobiology, are likely to reflect on global patterns. Longitudinal observations are needed to better characterize the state or trait nature of topological brain alterations, and their progression in different stages of the disorder. In this regard, the possibility of recruiting experimental samples that are homogeneous in the age of onset and in illness duration could be particularly useful. Moreover, given the paucity of data on patients with BN, further evidence on this disorder should be provided.

The main limitation of the present review is the lack of homogeneity in the designs and methods of the included studies, which conducted graph theory analyses applying different methodologies for constructing and analyzing brain networks and using a heterogeneous array of topological descriptors that cannot be directly compared. These issues (partly inherent to the novelty of the methods) prevent the possibility of conducting a meta-analysis of these data. The strengths of this study lie in the fact that it provides the first systematic overview of connectomic neuroimaging analyses in EDs. Moreover, it allows identifying both points of interest and critical issues of applying these tools to date, thus helping to direct future research. Specifically, a systematic overview of the designs and analyses present in the literature could help foster the homogeneity in the employed methodologies that is now lacking, impairing proper comparability of the results.

### What is already known about this topic?

The application of graph theory tools to the analysis of both structural and functional neuroimaging data in EDs is still at its infancy, but it has proven to have great potential for describing network-wise unbalances in the architecture of regional interrelations. At present, the results of connectomic analysis in EDs evidenced the presence of alterations in integration and segregation properties of the brain that take different directions based on imaging modalities (surface-based cortical analysis, DTI, fMRI) and to the stability of covariance networks to specific pathophysiological mechanisms.

### What this study adds

This study highlights the importance of investigating the rules that govern the relationships between different brain structures in EDs using a connectomics approach. These tools have displayed relevant cross-methodological patterns of alterations but need more systematic investigation. Moreover, the review underlines the importance of implementing these kinds of analyses with multimodal imaging protocols and longitudinal designs.
